# Exogenous Melatonin Improves Tolerance to Water Deficit by Promoting Cuticle Formation in Tomato Plants

**DOI:** 10.3390/molecules23071605

**Published:** 2018-07-02

**Authors:** Fei Ding, Gang Wang, Meiling Wang, Shuoxin Zhang

**Affiliations:** 1 College of Forestry, Northwest A&F University, Yangling, Shaanxi 712100, China; feiding82@gmail.com (F.D.); wang20180823@sina.com (G.W.); wangmeiling2081@163.com (M.W.); 2 Guizhou Academy of Forestry, Guiyang, Guizhou 550005, China

**Keywords:** melatonin, cuticle, cutin, cuticular wax, water deficit, leaf permeability, tomato plants

## Abstract

The plant cuticle, composed of cutin and waxes, is a hydrophobic layer coating the aerial organs of terrestrial plants and playing a critical role in limiting water loss. While melatonin has been recently demonstrated to be involved in responses to drought stress in plants, its relationship with cuticle formation is not known. In the present work, we report the effects of melatonin on the formation of cuticle in tomato leaves subjected to water deficit. Preliminary analysis by light microscope showed that tomato leaves pretreated with exogenous melatonin might have thicker cutin than tomato leaves without melatonin pretreatment under water deficit condition. Chemical characterization showed that exogenous application of melatonin increased the level of cuticular waxes in tomato leaves under water deficit. Consistent with the change in cuticular waxes was the increased abundance of wax-associated gene transcripts. Further, assessment of water loss and chlorophyll leaching in tomato leaves revealed the association of cuticle deposition with reduced leaf permeability, which is important in restricting water loss in water deficit-stressed tomato plants. These results suggest a role for melatonin in regulating leaf cuticle formation and non-stomatal water loss in leaves.

## 1. Introduction

The plant cuticle is a hydrophobic layer covering the outermost surfaces of terrestrial plants. The cuticle consists of cutin and cuticular waxes. Cutin is a polymer of ester-linked ω-hydroxylated fatty acids, while cuticular wax is a complex mixture of very-long-chain fatty acids (VLCFAs), VLCFA derivatives and secondary metabolites, such as triterpenoids and flavonoids [1,2,3]. As it represents the interface with plants and the surrounding environment, the cuticle plays a wide range of roles in plant development and physiological responses to biotic and abiotic stresses. One of the critical functions of cuticle is to control non-stomatal transpirational water loss [4,5]. The cuticle also confers protection in plants against excessive UV radiation, pathogen attack and insect herbivores [6,7]. In addition, the cuticle plays a central role in defining organ boundaries during development as demonstrated in tomato mutant deficient in cuticular wax [8].

The regulation of cuticle formation is complex and is further complicated by compensatory mechanisms between cutin and wax biosynthesis. The cuticle synthesis is influenced by a variety of environmental factors, including water deficit, light, temperature and moisture. Besides, signaling networks involved in responses induced by these environmental cues may contribute to the cuticle synthesis [9,10]. Furthermore, as the cuticle is exclusively formed in epidermis cells, factors affecting the development of epidermis may also influence cuticle synthesis [11]. Water deficit, one of the common abiotic stresses, has been reported to induce the synthesis of cuticular wax and cutin monomers in plants [12,13]. The key response to water deficit in plants is the accumulation of phytohormone abscisic acid (ABA), and it has been suggested that the cuticle composition and associated gene expression are altered by ABA, supporting a regulatory role of ABA in cuticle formation [12].

Melatonin (*N*-acetyl-5-methoxytryptamine) is an extensively-studied hormone playing important roles in a variety of biological processes in animals. Melatonin in plants was first identified by two research groups in 1995 [14,15]. In recent years, the molecule has received widespread attention for its versatile functions in plants. Melatonin is well known as an antioxidant molecule involved in the mitigation of oxidative stress either by directly scavenging ROS or indirectly improving antioxidant potential [16,17]. Melatonin has also been documented to improve tolerance to multiple stresses in plants, including cold, heat, salinity, drought, heavy metal toxicity and pathogens [18,19,20,21,22,23]. In addition, melatonin has been found to play a role in the delay of senescence in plants [24,25]. In the past few years, despite the significant progress that has been made in elucidating the role of melatonin in abiotic stress tolerance in plants, the mechanisms of melatonin-mediated drought tolerance are still not fully understood and merit further investigation.

In this work, to address the hypothesis that melatonin mediates cuticle formation during water deficit, we investigated the effects of exogenous melatonin on cuticle formation by assessing the cutin structure and cuticular wax content in tomato leaves under water deficit stress. We also determined the expression of genes involved in the cuticular wax biosynthetic pathway. Finally, we measured the rates of water loss and chlorophyll leaching in tomato leaves to infer changes in cuticle permeability as affected by exogenous application of melatonin.

## 2. Results

### 2.1. Phenotypes of Tomato Plants Treated with or without Melatonin under Water Deficit Condition

**Figure 1:**
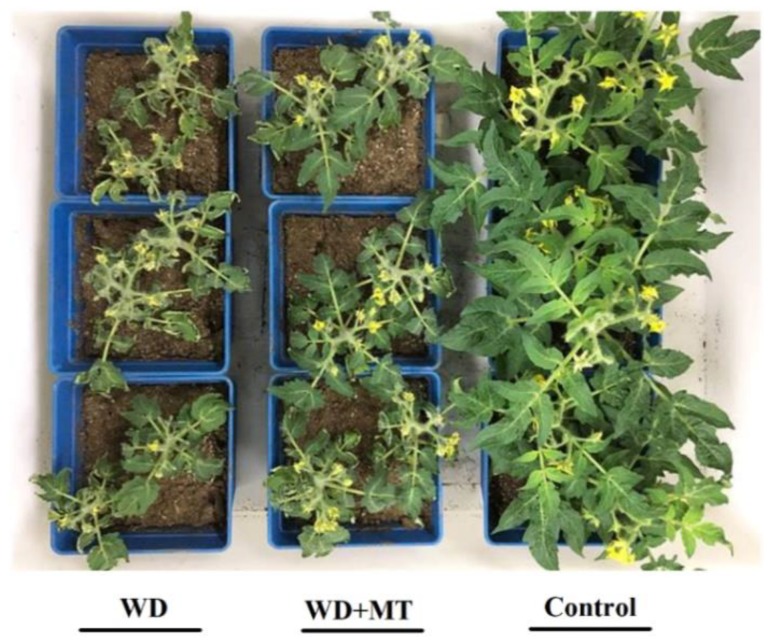
Phenotypes of Water Deficit-stressed tomato plants (WD), water deficit-stressed tomato plants with Melatonin pretreatment (WD + MT) and well-watered tomato plants (Control). At the five-leaf stage, tomato plants were sprayed with 100 μM melatonin one time a day on three consecutive days, and then, plants were deprived of water for 14 days.

Melatonin has been reported to play a role in conferring drought tolerance in plants [26,27]. We observed that compared with that in well-watered plants, the growth was severely retarded and leaves appeared wilted in water deficit-stressed tomato plants. However, growth inhibition of tomato plants under water deficit was partially relieved by exogenous application of melatonin (Figure 1), in accordance with previous studies showing that exogenous melatonin alleviated drought stress in plants [20,28,29].

### 2.2. Effect of Exogenous Melatonin on Leaf Cutin under Water Deficit Condition

**Figure 2:**
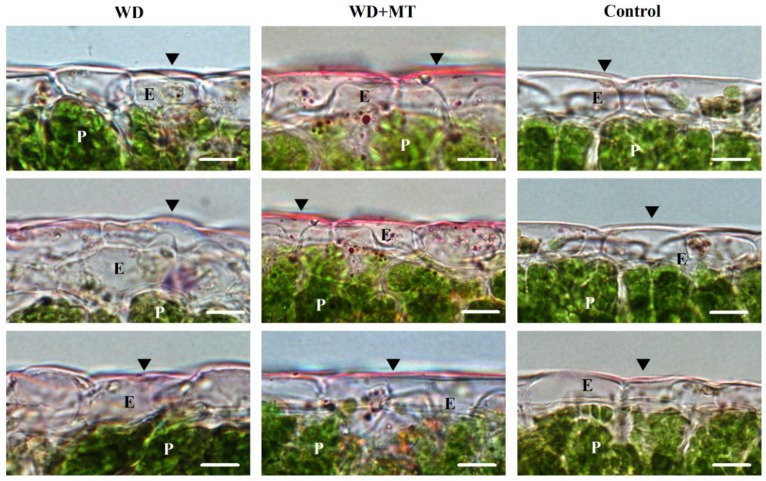
Effect of exogenous melatonin on leaf cutin under water deficit condition. Light micrographs showing the leaf cuticle cutin layer stained with Sudan Red (arrowheads). E, Epidermal cell; P, Palisade cell. Scale bar = 10 μm. At the five-leaf stage, tomato plants were sprayed with 100 μM melatonin one time a day on three consecutive days, and then, plants were deprived of water for 10 days. Leaf samples were collected from different groups at Day 10 following water deprivation for cutin analysis.

Cutin is one of the two components of leaf cuticle. To assess the effect of exogenous melatonin on leaf cutin, leaf cross-sections from tomato plants pretreated with or without melatonin under water deficit were stained and viewed under light microscope. The leaf cutins from water deficit plants were thicker than those from well-watered plants, but thinner than those from melatonin-pretreated plants under water deficit stress (Figure 2), indicating that melatonin promoted cutin synthesis under water deficit condition.

### 2.3. Exogenous Melatonin Enhances Leaf Cuticular Wax Accumulation under Water Deficit Condition

**Figure 3:**
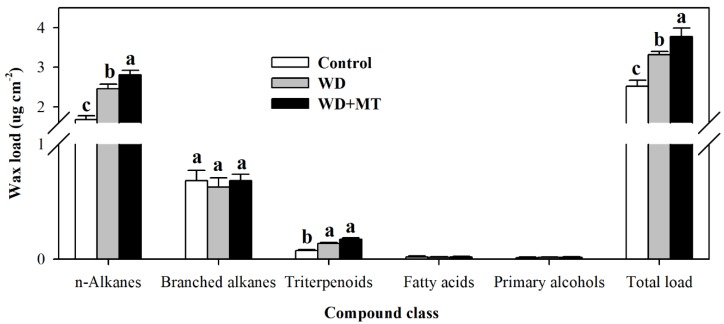
Cuticular wax accumulation in tomato leaves pretreated with or without melatonin under water deficit stress. Cuticular waxes were extracted and analyzed using GC-MS. At the five-leaf stage, tomato plants were sprayed with 100 μM melatonin one time a day on three consecutive days, and then, plants were deprived of water for 10 days. Leaf samples were collected from different groups of plants at Day 10 following water deprivation. The values presented are the means ± SDs (*n* = 3). Different letters indicate significant differences at *p* < 0.05 among treatments.

Cuticular wax is a major constituent of leaf cuticle. To understand the role of melatonin in the biosynthesis of cuticular wax, wax loads and wax compositions were determined. The results showed that total wax loads in non-melatonin-treated plants and melatonin-treated plants under water deficit stress were significantly enhanced in comparison with that in normally-grown tomato plants, and the enhancement was more pronounced in melatonin-treated plants under drought stress (Figure 3). Further analysis showed that the dominant wax component in tomato leaves was alkane, and the melatonin-mediated increase in total wax was due to the change in alkane content.

### 2.4. Exogenous Melatonin Promotes the Expression of Wax Biosynthetic Genes

**Figure 4:**
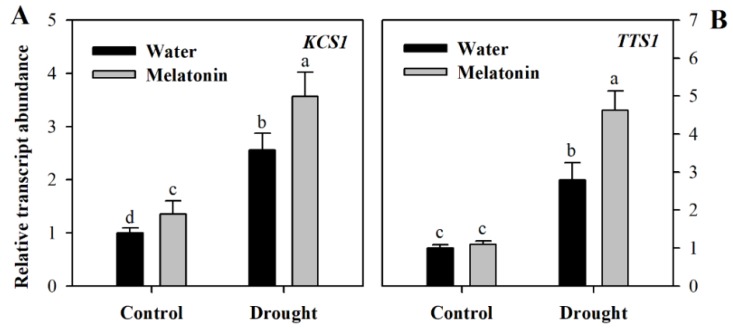

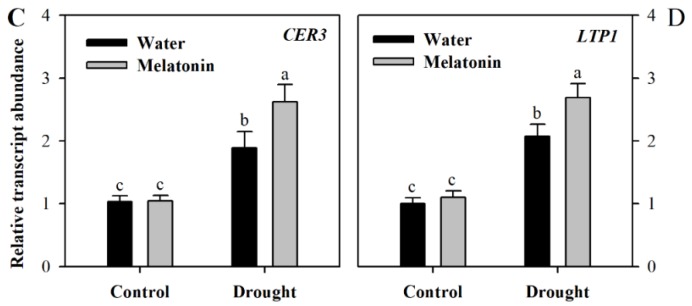
Relative transcript abundance of genes involved in wax biosynthesis in tomato plants treated with or without melatonin. (**A**) *KCS1*; (**B**) *TTS1*; (**C**) *CER3*; (**D**) *LTP1*. Results were presented as fold change relative to control plants sprayed with distilled water. The relative transcript abundance was determined by quantitative real-time PCR. At the five-leaf stage, tomato plants were sprayed either with 100 μM melatonin or distilled water one time a day on three consecutive days, and then, plants were deprived of water for 10 days. Leaf samples were collected from different groups of plants at Day 10 following water deprivation. The values presented are the means ± SDs (*n* = 3). Different letters indicate significant differences at *p* < 0.05 among treatments.

To gain further understanding of the impact of melatonin on cuticular wax biosynthesis, we evaluated the relative expression of genes involved in wax biosynthesis. These genes included *KCS1* involved in the elongation of fatty acids (Gene ID: Solyc10g009240.2, ketoacyl-CoA synthase), *CER3* responsible for the synthesis of alkane (Gene ID: Solyc03g117800.2, very-long-chain alkane synthase), *TTS1* involved in the synthesis of the triterpenoids (Gene ID: Solyc12g006530.1, beta-amyrin synthase/oxidosqualene cyclase) and *LTP1* responsible for the transport of lipids (Gene ID: Solyc10g075070.1 non-specific lipid-transfer protein,). The relative transcript abundance was determined using leaf samples collected from different groups of plants at Day 10 following water deprivation. It was observed that the expression of four wax biosynthetic genes was induced by drought stress and was further increased in response to exogenous melatonin (Figure 4), indicating the role of melatonin in promoting wax biosynthesis. Under well-watered condition, exogenous application did not influence the expression of wax biosynthetic genes much at Day 10 following exogenous spray of melatonin.

### 2.5. Melatonin-Mediated Increase in Cuticle Formation Reduces Leaf Permeability

**Figure 5:**
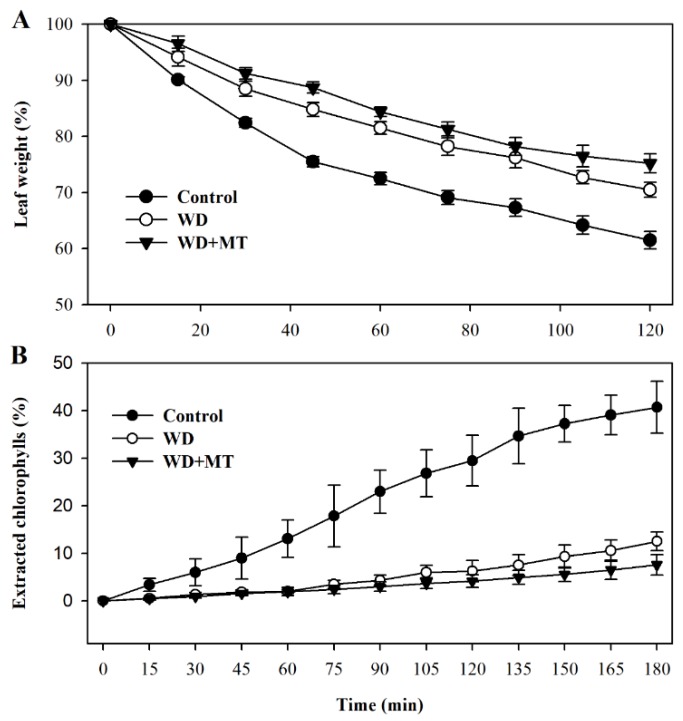
Water loss rates and chlorophyll leaching rates of tomato leaves pretreated with or without melatonin under water deficit stress. (**A**) Water loss rates were expressed as a percentage of initial water saturated weight; (**B**) chlorophyll leaching rates were expressed as a percentage of total chlorophyll extracted. Samples were harvested from tomato plants at Day 10 following water deprivation or from well-watered plants.

Leaf permeability affects non-stomatal water loss in leaves. In order to examine more directly whether the melatonin-mediated increase in cuticle formation is linked with water loss in tomato plants, we assessed leaf permeability by measuring cuticular evaporation. The results showed that cuticular evaporation was slower in leaves of plants treated with melatonin and water deficit than that in leaves of plants treated with only water deficit (*p* < 0.05 at 45 min, 60 min, 75 min, 105 min and 120 min; Figure 5A), consistent with the increased accumulation of cuticular wax in melatonin-treated plants under water deficit condition.

To further ascertain the linkage between cuticular wax accumulation and control of non-stomatal water loss, we examined chlorophyll leaching in leaves subjected to different treatment. In agreement with cuticular evaporation, chlorophyll leaching was slower in leaves of tomato plants treated with melatonin under water deficit stress (*p* < 0.05 at 150 min, 165 min and 180 min; Figure 5B). These results demonstrate that melatonin-mediated cuticular wax accumulation may contribute to reduced water loss in tomato plants.

## 3. Discussion

The plant cuticle is a ubiquitous layer of land plants, which plays a crucial role in restricting non-stomatal water evaporation. Studies have demonstrated that the increase in cuticles, consisting of cutin and cuticular wax, reduces water loss in leaf [12,13]. The formation of cuticles can be influenced by a number of factors, including light, temperature, moisture and hormones [9,30]. In the present study, we found that melatonin, an extensively-studied molecule, played a new role in tomato plants exposed to water deficit. We concluded that exogenous melatonin promoted cuticle formation and thus reduced leaf permeability in water deficit-stressed tomato plants. The evidence leading to the conclusion includes: (1) pretreatment with melatonin might lead to thicker leaf cuticle in water deficit-stressed tomato plants; (2) exogenous melatonin increased total leaf cuticular wax load in water deficit tomato plants; (3) exogenous melatonin increased the expression of some wax biosynthetic genes; (4) pretreatment with melatonin reduced leaf permeability.

In recent years, melatonin has been demonstrated to play a wide range of roles in development and responses to abiotic and biotic stresses in plants. There have been several reports on the influence of melatonin on drought resistance in plants [19,20,26,27]. In the present work, we also observed that exogenous application of melatonin improved tolerance to water deficit stress in tomato plants. However, instead of focusing on the widely-reported role of melatonin in detoxifying ROS in drought-stressed plants, we sought to explore new possible roles of this molecule.

Unlike previously-reported roles of melatonin, we found that it might function in the formation of leaf cuticles by affecting the biosynthesis of cutin and cuticular waxes. The analyses of leaf cuticles from water deficit, water deficit plus melatonin-pretreated and well-watered tomato plants indicated that melatonin is involved in the regulation of leaf cuticles. Exogenous application of melatonin might induce thicker cutin in water deficit tomato plants in comparison with control plants and water deficit plants without melatonin application, suggesting that this molecule may promote the synthesis of the leaf cuticles in water deficit-stressed tomato plants. Further analysis showed that the levels of cuticular waxes, a key cuticle component, were significantly enhanced in melatonin-pretreated tomato plants under water deficient condition. Consistent with the increased wax accumulation was the enhanced expression of genes involved in wax biosynthesis under water deficit. We observed that at Day 10 following melatonin pretreatment that the expression of four wax biosynthetic genes, *KCS1*, *CER3*, *TTS1* and *LTP1*, was induced by drought stress and was further increased in response to exogenous melatonin, whereas exogenous application did not influence the expression of wax biosynthetic genes much under well-watered condition. The rise in wax accumulation, together with increased induction by melatonin of wax biosynthetic genes, highlights the regulatory role of melatonin in wax biosynthesis in tomato leaves.

Previous studies on rose (*Rosa* × *hybrida*) [31], tree tobacco (*Nicotiana glauca*) [32] and *Arabidopsis* [33] have shown that the increase in wax accumulation leads to a less water-permeable cuticle, allowing limiting of transpiration and delaying of leaf wilting under drought conditions. The results presented in this study showed that melatonin promoted the biosynthesis of leaf cuticular waxes under water deficit stress, which is thought to be associated with the less permeable cuticles, as demonstrated by reduced cuticular evaporation and chlorophyll leaching. Melatonin-mediated increase in cuticular waxes and the resulting decreased cuticle permeability may contribute to the phenotypes we observed in Figure 1. Though antioxidant capacity influenced by melatonin in tomato plants has not been examined in the current work, it is accepted that melatonin acts as an important molecule in conferring enhanced antioxidant potential in plants exposed to environmental stresses, and this action has been verified in a great number of studies. Thus, it is likely that the improved tolerance to water deficit stress in tomato plants might also be ascribed to antioxidant functions of melatonin. As observed in the work of Lazar et al., exogenous melatonin increased photosynthetic performance by protecting the photosynthetic proteins from oxidative damages [34]. Enhanced photosynthetic capacity enables plants to have more energy to cope with environmental stresses.

However, Figure 2 in this study has to be cautiously interpreted. Light microscopy images may not provide enough resolution to accurately judge the thickness of cutin, probably resulting in the devaluation of the role of melatonin in cutin biosynthesis. Use of transmission electron microscopy may help solve the problem. There is another point that warrants attention in this study. In the examination of chlorophyll leaching rate, the duration of 180 min of bleaching may not be long enough to distinguish the cuticle permeability assessed by chlorophyll leaching. For follow-up experiments on chlorophyll leaching of tomato leaves, 2 h of leaching is suggested.

In summary, we have obtained preliminary results that support a previously unknown role of melatonin. We found that melatonin promoted leaf cuticle formation in water deficit-stressed tomato plants. The increased cuticles led to reduced permeability, consequently restraining non-stomatal water loss and likely delaying leaf wilting in the period of water deficit. However, as an issue that requires further study, the underlying mechanism of the regulatory roles of melatonin in cuticle formation remains yet unclear.

## 4. Materials and Methods

### 4.1. Plant Materials, Growth Conditions and Treatment

Tomato (*Solanum lycopersicum* L. cv. Micro-Tom) seeds were sterilized and germinated at 25 °C in the dark on filter paper in Petri dishes. Germinated seeds were then planted in 12 cm × 12 cm plastic pots containing peat and vermiculite (3/1, *v*/*v*) and maintained in a growth room with the following conditions: 380 ppm of CO_2_, photon flux density of 400 μmol m^−2^ s^−1^, day/night temperature of 25/20 °C, relative humidity of 60% and a photoperiod of 16 h.

At the 5-leaf stage, tomato plants were sprayed one time a day either with 100 μM melatonin (Sigma-Aldrich, St. Louis, MO, USA) solution or with distilled water for 3 consecutive days, resulting in two groups of plants. Then, plants sprayed with distilled water were randomly split into two groups, one of which received regular watering management (control), and the other was deprived of water for two weeks (Water Deficit (WD)). Plants pretreated with melatonin were also deprived of water for two weeks (Water Deficit + Melatonin (WD + MT)). For each repeat of the experiment, there were a total of 15 tomato plants, and each of three replicates consisted of 5 plants.

Samples of mature leaves were collected at 6 h and 12 h following the last spray of melatonin for analysis of gene expression. Leaf samples were collected at Day 10 following water deprivation for wax analysis and leaf permeability assay.

### 4.2. Analysis of Cutin Layer

The analysis of cutin was performed following the protocol by Yeats et al. (2013) [35]. Leaf cross-sections from each group were used to analyze the difference of the cutin layer. The cross-sections were stained with Sudan Red in a humidity chamber for 1 h, then washed and mounted with 75% glycerol. The stained slides were viewed on a bright field microscope (Olympus BX51 + DP70, Tokyo, Japan) using 100× oil immersion objectives.

### 4.3. Analysis of Cuticular Wax

Total wax load and chemical compositions were analyzed according to Wang et al., (2017) with minor modifications [36]. The analysis was performed by GC-MS and GC-FID. Tomato leaves were harvested and immersed in chloroform for 60 s to extract waxes. Extracted wax samples were dried and derivatized with equal amounts of pyridine (Alfa Aesar) and BSTFA (*bis*-*N*,*O*-(trimethylsilyl) trifluoroacetamide) (Fluka) for 40 min at 70 °C. Then, samples were dried using nitrogen gas and were re-dissolved in chloroform. *n*-tetracosane (C24) was added into samples as an internal standard. Wax compounds were quantified by gas chromatography equipped with a mass spectrometric detector (GC-MS) (GCMS-QP2010, SHIMADZU, Japan). The content of wax component was calculated using peak area in comparison with that of the C24 internal standard.

### 4.4. Determination of Transcript Abundance of Wax Biosynthetic Genes by Quantitative Real-Time PCR

Total RNA was extracted from tomato leaves and was used for cDNA synthesis by PrimeScript^®^ reverse transcriptase following standard protocols. Quantitative real-time PCR was performed using SYBR^®^ Premix Ex TaqTM (TaKaRa) according to the manufacturer’s instructions. Each real-time PCR reaction was performed in a 25-µL final volume on an iQ5 Multicolor Real-Time PCR Detection System (BIO-RAD, USA) under the following program: 1 cycle of 30 s at 95 °C, followed by 40 cycles of 5 s at 95 °C and 30 s at 60 °C. The primers used in this study: CTGGTCCACACTGTTCGAACT (forward) and AAGTTCACGAGCTAGTGACACC (reverse) for *KCS1* (ketoacyl-CoA synthase, Solyc10g009240.2), ATATCACTGGCATCACCACGAG (forward) and CGTAACCAATAAATGCAGTGCCT (reverse) for CER3 (very-long-chain alkane synthase, Solyc03g117800.2), ACAGGCAACTGATGGTCATT (forward) and CCATCTTCGTTCTGATGACAG (reverse) for *TTS1* (beta-amyrin synthase, Solyc12g006530.1) and GTTCAGTAATTCGAGCAGTCAGT (forward) and CCTCAAATCAGCTGCTAATGCT (reverse) for *LTP1* (non-specific lipid-transfer protein, Solyc10g075070.1) and CTTGTCTGTGACAATGGAACTG (forward) and ATACCCACCATCACACCAGTAT (reverse) for internal reference actin gene (GenBank Accession No. AB695290).

### 4.5. Measurement of Epidermal Permeability

Epidermal permeability was assessed according to Kosma et al. (2009) with minor modifications [12]. In short, to quantify non-stomatal water loss, tomato plants were dark acclimated for 6 h prior to measurement to make sure that stomata were fully closed. Then, the whole shoot was excised and placed immediately in water in the dark and soaked for 90 min to completely rehydrate leaves. Then, water-saturated leaf weights were determined and recorded every 15 min using a microbalance. Data were expressed as a percentage of the initial fresh weight.

Epidermal permeability was also assessed using chlorophyll leaching. One gram of leaves in each group was immersed in glass tubes with 50 mL of 95% ethanol at room temperature in the dark. The amount of chlorophyll extracted in the solution was quantified by measuring absorbance at 647 and 664 nm using a spectrophotometer every 15 min after immersion. The total chlorophyll amount of each sample was determined after 48 h since the initial immersion. Data were expressed as a percentage of the total chlorophyll amount.

### 4.6. Statistical Analysis

All experiments were repeated at least three times in this study, and the values presented are the means ± SDs. Duncan’s multiple range test was performed to compare the difference among treatments. Different letters in figures indicate significant differences at *p* < 0.05.
